# The process of developing a joint theory of change across three global entities: can this help to make their efforts to strengthen capacity for implementation research more effective?

**DOI:** 10.1136/bmjph-2023-000029

**Published:** 2024-03-19

**Authors:** Garry Aslanyan, Kabir Sheikh, Marta Feletto, Pascal Launois, Mahnaz Vahedi, Vanessa Brizuela, Anna Thorson, Sara Begg, Susie Crossman, Imelda Bates

**Affiliations:** 1 UNICEF/UNDP/World Bank/WHO Special Programme for Research and Training in Tropical Diseases (TDR), WHO, Geneva, Switzerland; 2 University College London, London, UK; 3 Alliance For Health Policy and Systems Research, WHO, Geneva, Switzerland; 4 UNDP/UNFPA/UNICEF/WHO/World Bank Special Programme of Research, Development and Research Training in Human Reproduction (HRP), Department of Sexual and Reproductive Health and Research, WHO, Geneva, Switzerland; 5 Centre for Capacity Research, Liverpool School of Tropical Medicine, Liverpool, UK

**Keywords:** Public Health, Community Health, Social Medicine

## Abstract

**Introduction:**

A theory of change is a visual representation of the pathway by which a programme anticipates it will achieve its goal. It usually starts with discussions around the goal and works backwards through outcomes and outputs to activities.

**Methods:**

We used a theory of change to improve coherence across three research entities at the WHO. Part of the remit of all three entities is to strengthen capacity in low-income and middle-income countries for implementation research.

**Results:**

Representatives from the three entities were able to formulate a joint goal for strengthening capacity in implementation research. They identified three pathways by which this could be achieved: (a) conducting implementation research, (b) strengthening implementation research systems and (c) using implementation research for public health priorities.

**Conclusion:**

The process of developing the theory of change and the logic framework it created, provided a means to track progress towards the goal and to guide improvements in programmes within their lifetime. The process we used to develop the theory of change and the pathways to achieve the joint goal are adaptable and could be used by other organisations that also aim to strengthen research capacity. This would lead to more coherence, better translation of research findings into decision-making and ultimately improvements in public health.

**WHAT IS ALREADY KNOWN ON THIS TOPIC**

- Implementation research (IR)—the systematic study of methods to apply research findings to policy and practice—requires particular skills and has traditionally been overlooked in favour of less applied types of research.
- Three research entities at the WHO and focused on health systems, sexual and reproductive health and rights, and infectious diseases of poverty, all conduct activities to strengthen capacity for IR, primarily in low-income and middle-income countries.
- Global efforts to strengthen IR capacity need to be accelerated since enhancing nations’ ability to undertake research and to use findings for policy or practice is essential for evidence-based decision-making for development and democracy.
- A theory of change is a useful tool to guide the evaluation of complex health interventions as it can map out the flow pathway from activities and outputs, to outcomes and impact and help to elucidate why and how change happens.
- Although theories of change are beginning to be used by researchers and also by some health research funders, there is very little published information about their use in programmes that aim to strengthen capacity for IR.

**WHAT THIS STUDY ADDS**

- Creating a joint theory of change was useful for identifying common pathways for achieving strengthened capacity for IR. These pathways focused on (a) conducting IR, (b) strengthening IR systems and (c) using IR for public health priorities and would be a useful foundation for other programmes with similar goals.
- Through the development of a theory of change, we were able to disentangle and understand the themes across all three programme that were relevant for IR capacity strengthening, and to identify the opportunities and pathways for joint activities across these complex global programmes.
- The process of jointly developing the theory of change and the framework it created, provided a means to track activities over time, and also demonstrated how the three entities’ efforts and networks could be rapidly repurposed in response to national needs during the COVID-19 pandemic.

**HOW THIS STUDY MIGHT AFFECT RESEARCH, PRACTICE OR POLICY**

- A joint theory of change is a useful way to better understand how global research programmes can together be more effective in strengthening capacity for IR.
- The process of developing a joint theory of change is an effective way to align programmes’ efforts around well-defined pathways to impact and can be used to promote coherence in activities and goals. It can be applied across complex programmes, even when they are well established, and facilitates adjustments within a programme’s lifetime.
- Our theory of change is adaptable and potentially transferable to other international agencies and non-governmental organisations involved in strengthening research capacity, ultimately leading to better decision-making and health improvements.

## Introduction

 The importance of implementation research (IR) is growing among international health programmes because it advances understanding about implementation strategies and mechanisms, and the enablers and barriers to implementing evidence-based policy and practice, and promotes the translation of research into benefits for public good.^1^ IR is underpinned by theories and analytic frameworks to ensure research quality and rigour.^2^ It is particularly useful for evaluating the process of complex health interventions.^3^

Three diverse and well-established global programmes at the WHO are all committed to developing capacity for IR although this is not necessarily their main focus. They work primarily through global partnerships and by facilitating their partner organisations and institutions to undertake complex health interventions and to strengthen partners’ IR capacity. These partners are primarily university departments and research institutions but also include public health agencies, public health or research departments of Ministries of Health, research or medical research councils in countries, non-governmental organisations and delivery organisations that conduct IR to help in implementation of their programmes. Other partners include UN agencies in countries, such as UNICEF, UNFPA, World Bank, WHO and UNDP. Each programme has its own research interest: the Alliance for Health Policy and Systems Research^4^ (AHPSR) supports the generation and use of evidence on important health systems challenges, and research capacity building to strengthen health systems in low-income and middle-income countries (LMICs); the UNDP/UNFPA/UNICEF/WHO/World Bank Special Programme of Research Development and Research Training in Human Reproduction Program^5^ (HRP) focuses primarily on sexual and reproductive health and rights research and research training; and UNICEF/UNDP/World Bank/WHO/Special Programme for Research and Training in Tropical Diseases^6^ (TDR) focuses on IR methods primarily applied to infectious diseases of poverty.

Our aim was for all three entities to develop a joint theory of change to help them better understand how together they can be more effective in strengthening capacity for IR in LMICs. A theory of change is a diagrammatic description of how a desired change is expected to happen to reach a specific goal and is a methodology for planning, participation, management and evaluation to promote change.^7^ A strength of a theory of change compared with, for example, a logical framework, is that it encompasses, and therefore, promotes discussion about, how and why the expected change will be brought about.

## Materials and methods

### Developing the joint theory of change

Usually, a theory of change is developed in the context of a single new programme. For example, the National Institute of Health Research UK have developed a theory of change that illustrates how its Global Health Research programme aims to bring about its intended outcomes and impacts.^8^ A theory of change starts with identifying the goal of a programme and then working backwards to identify the anticipated outcomes and activities needed to achieve impact. However, in our case, the theory of change (including its vision and goal) had to be aligned to the existing strategies of the three programmes: each strategy was underpinned by the Sustainable Development Goals (SDGs).^9^ The first step was, therefore, to determine the programmes’ joint vision and goal for their research capacity strengthening (RCS) efforts. After that, activities related to strengthening capacity for IR within each programme were identified and then the activities were reviewed and used to formulate joint outcomes and ultimate goals. Since all three programmes already had their own well-established activities and workplans, we adapted the traditional process for developing a theory of change for a new programme, so we focused especially on identifying the pathways that would lead to achieving their common goal of strengthening IR capacity. The programmes each provided documents that provided more details about each of their activities (online supplemental table S1). A joint theory of change was developed by two to three representatives from each of the three programmes (seven participants in total). They were selected to participate in the study because they were senior leaders and managers in their programmes with good overall knowledge and understanding of, and influence over, their programmes. They participated in a 2-day workshop facilitated by two external consultants with experience in developing theories of change. No patients or members of the public were involved in the design, conduct or reporting of this study.

### Articulating the joint vision and goal

**Figure 1:**
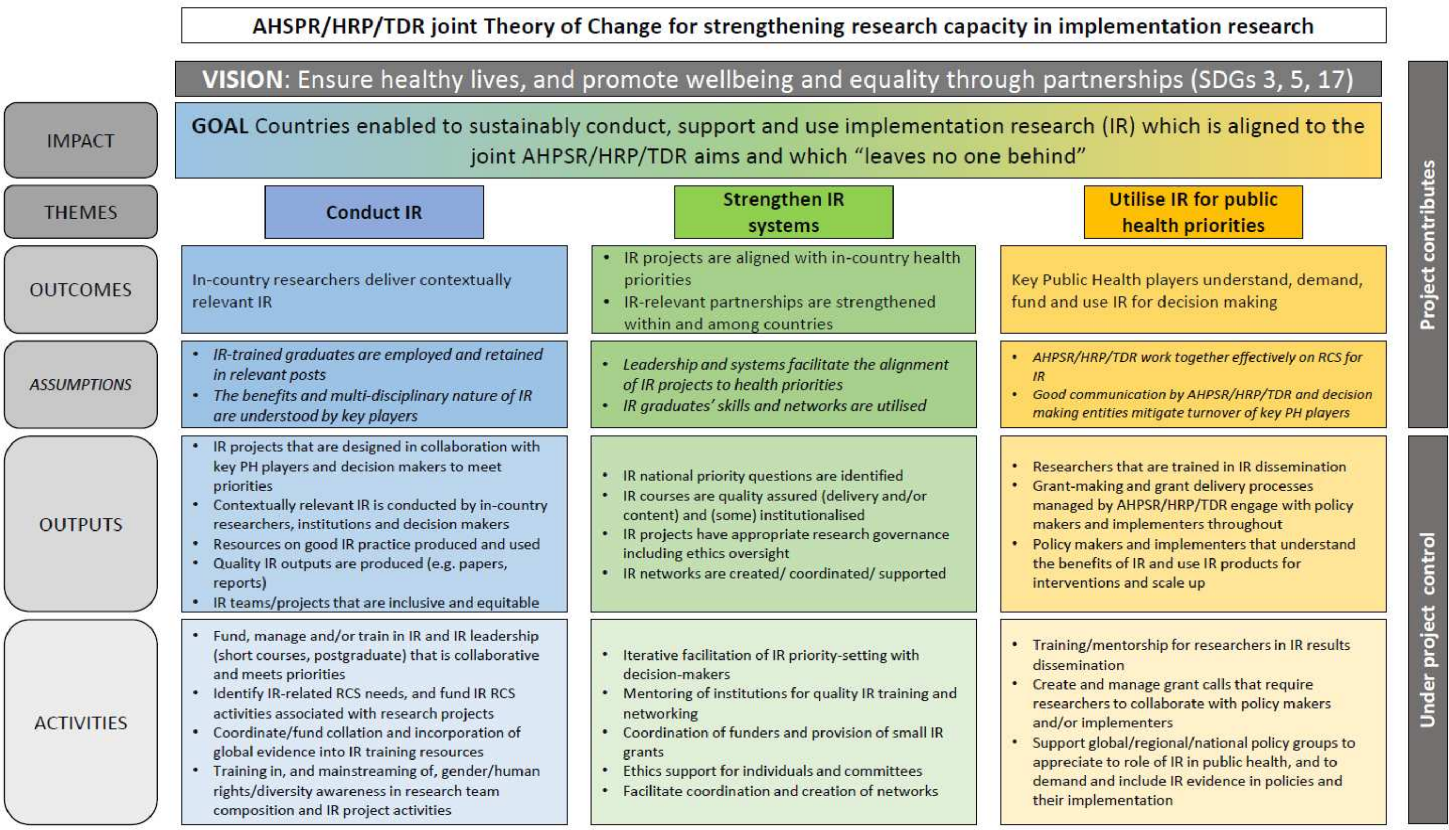
Theory of change for strengthening capacity in IR across three global health entities (AHPSR, HRP, TDR). AHPSR, Alliance for Health Policy and Systems Research; HRP, the UNDP/UNFPA/UNICEF/WHO/World Bank Special Programme of Research Development and Research Training in Human Reproduction; IR, implementation research; PH, public health; RCS, research capacity strengthening; SDG, sustainable development goal; TDR, UNICEF/UNDP/World Bank /WHO/ Special Programme for Research and Training in Tropical Diseases.

An iterative process was adopted starting with achieving agreement on draft wording for a single goal for all three programmes for strengthening IR capacity. Participants agreed that the vision for the theory of change should be rooted in the SDGs since these were the foundation for all the programmes. The programmes were especially aligned to SDGs 3 and 5 (ensure healthy lives and promote well-being for all at all ages; achieve gender equality and empower all women and girls respectively) and SDG17 (strengthen the means of implementation and revitalise the global partnership for sustainable development) since all the programmes’ activities, together and independently, were based on partnerships and intersectoral approaches. The final vision incorporated into the theory of change (figure 1)—‘ensure healthy lives, and promote well-being and equality through partnerships’—was therefore an amalgamation of these three SDGs.

The goal of the theory of change needed to align to the programmes’ existing strategies and to the WHO General Work Programme 13 which reflected two key principles that were important to the programmes—RCS and the role of knowledge translation, and the interface between research and policy.^10^ To reflect the vision of promotion of equality, the programmes’ representatives also incorporated the ‘leave no one behind’ approach.^11^ This aims to ‘eradicate poverty in all its forms, end discrimination and exclusion, and reduce the inequalities and vulnerabilities that leave people behind and undermine the potential of individuals and of humanity as a whole’. The final goal, which combined these perspectives and was also orientated towards IR, was ‘countries enabled to sustainably conduct, support and use IR which is aligned to the joint AHPSR/HRP/TDR aims and which "leaves no one behind" ’. This goal assumes that if countries can sustainably conduct, support and use IR, and if this research ‘leaves no one behind’ by considering how interventions impact on different groups within the population, the result will be an increased uptake of effective interventions in these countries. Underpinning this was an assumption that these interventions would in turn contribute to improved systems and health and well-being across the population (i.e. the vision).

### Identifying each programme’s RCS IR activities and the pathways by which they lead to the outcomes

During the workshop, each programme described all their current activities in strengthening capacity for IR and the outcomes from these activities and categorised these according to the constituent parts of the goal. These activities fell into three broad pathways linking activities with the outcomes, goal and vision. These pathways were refined during workshop discussions and any disagreements and refinements were resolved through dialogue among all the participants. The pathways formed the basis of the joint theory of change (figure 1) and were: conducting IR, strengthening IR systems and utilising IR for public health priorities.

These pathways were interconnected and did not represent a linear process. For example, to achieve the outcomes related to the utilisation of IR, policy-makers need to also have their capacity to understand IR strengthened. A final phase of discussion considered factors outside the programmes’ control (i.e. ‘assumptions’) that might impact on their ability to achieve their goal. Examples included individuals trained in IR would be employed and retained in relevant posts, the benefits and multidisciplinary nature of IR were understood by key players, and the programmes would work together to achieve the goal of strengthening capacity for IR.

### Pathways within the theory of change

#### Conduct IR

Activities related to RCS on this pathway highlighted the role of training in IR as a cornerstone for building research capacity. Each programme engaged with training in slightly different ways, with variations in the funding and delivery of training, and in the development of resources for IR training. Training activities supported by the three programmes would mainstream the principles of equality and multidisciplinary participation in the RCS activities and in the research resulting from this capacity strengthening.

By conducting contextually relevant IR, researchers can generate evidence on how to enhance effectivity of proven interventions in real-life settings, with research questions tailored to the context. Skills, knowledge and funding are a prerequisite to conduct IR. Consequently, this pathway covered funding and training in collaborative, priority-led IR which mainstreams gender/human rights/diversity, collation of resources to support this training, and identifying and obtaining funding for IR-RCS activities. If these activities were delivered as intended, the resulting outputs would be IR projects designed to meet priorities that are conducted by inclusive and equitable teams of in-country researchers and institutions, and which result in quality IR outputs such as papers and reports. As a result of training and mainstreaming of gender, human rights and diversity, this would lead to equitable evidence generation and research outputs which ‘leave no one behind’.

#### Strengthen IR systems

The activities and outcomes in this pathway are diverse and reflect the way in which each programme has designed its activities to meet the needs of their different global partners. The activities across all three programmes fell into five categories—facilitating priority-setting of IR activities with decision-makers; mentoring of institutional staff to deliver high-quality IT training; provision of small IR grants; tools and processes for ethics reviews; and the creation and coordination of IR-related networks. Participants recognised that research systems must be in place to support the conduct of IR and for the research to be delivered effectively. Research systems are complex with multiple components, and therefore, the contribution of the three programmes defined in this theory of change was largely focused on the alignment of IR projects to research questions that are prioritised by stakeholders and the strengthening of IR partnerships.

The activities required to achieve this outcome were, therefore, driven by mentoring and support of, and networking between, key players in research systems including key decision-makers, institutions, ethics committees and funders. If these activities were delivered as intended the expected outputs included the identification of research priorities, institutionalised and/or quality-assured training, projects delivered with appropriate research governance and IR networks.

#### Utilise IR for public health

Similar to ‘research systems’ (above) the activities in this pathway reflect the responses of each programme to requests for support from their global members and partners. Participants described the need for policy-makers to routinely seek research evidence when making policy decisions. They also recognised that to make this research accessible to policy-makers, they needed to enhance knowledge translation skills among the IR community. Consequently, this pathway reflected these different ways key public health players interact with IR so it can be used effectively in policy-making. Participants anticipated that engaging key public health decision-makers in IR, and driving a culture of demanding, funding and using IR, would result in more useful IR being undertaken and ultimately to changes to practice which would contribute to the vision articulated in the joint theory of change.

Across all three programmes these activities fell into three categories—training in dissemination of IR findings; helping key players to understand IR, designing grant calls that require researchers to collaborate with policy-makers and/or implementers to further develop this understanding; and supporting policy groups to drive demand and use of IR in public health. If delivered as intended this would result in researchers that are trained in dissemination, and policy-makers that understand the outputs of IR and are engaged in the research throughout. It would also enhance the generic research skills of individuals and positively contribute to their academic careers and promotions.

### Using the theory of change to follow progress

After 18 months, the external facilitation team were able to use the joint theory of change as a framework against which to review changes in each programmes’ IR activities. Each programme provided documents (e.g. reports, journal articles, project briefs, website links) about their activities related to strengthening capacity for IR online supplemental table S1). The external team extracted information from these documents into a predesigned matrix based on the three pathways in the theory of change. At a joint workshop in 2021, the accuracy and interpretation of the extracted information was validated by the programmes and adaptations to their activities necessitated by the COVID-19 pandemic were also captured.

Prior to developing their joint theory of change, each programme had already created their own global networks of training centres (RCS regional hubs for HRP and technical support centres for TDR) mainly in universities. The programmes entrust these centres to deliver training on IR. The training provided by the programmes on IR and other topics is delivered face to face or, increasingly, online and provides a range of options from short courses to postgraduate degree programmes (i.e. master’s and doctoral).

All three entities incorporated activities on gender, human rights and diversity into training courses and toolkits and with partners. HRP had already partnered with AHPSR, TDR, Pan American Health Organisation (PAHO) and the HRP Alliance hub in the Americas to support research on sexual and reproductive health and rights and infectious diseases of poverty linked to mass migration in the Americas with a focus on RCS.

HRP and TDR embarked on assessing training needs regarding sex, gender and intersectionality. The three entities have jointly carried out needs assessments to determine the topics to be included in IR training courses and they all work to develop, communicate and support evidence-based policy and practice, and to promote capacity strengthening for IR through contribution and leadership in WHO guidelines (HRP), courses, frameworks, workshops and toolkits (AHPSR, HRP, TDR), by developing special journal issues (AHPSR, HRP) and academic publications (all).

To promote utilisation of implementation and other research, AHPSR and HRP insist that projects which they support must have relevance for and links to decision-makers or implementers. All three programmes have facilitated research priority setting in IR in their topic areas including through consultations and by providing supporting evidence such as peer-reviewed articles. Together programmes have provided support for IR projects on national priority topics through country-led IR programmes for universal health coverage (e.g. barriers to universal health coverage in Nepal).

### The impact of the COVID-19 pandemic on activities to strengthen capacity for IR

The global reach and networks of the three programmes meant they were all well positioned to rapidly respond with COVID-19-related research. Early in the pandemic, most of the programmes’ training and workshops, which were delivered through regional institutions, moved online, though in some countries this was only for a few weeks. Some students’ projects became desk- rather than field-based and others were reorientated to undertake COVID-19 research. The programmes, and their training partners, largely managed to overcome initial challenges with online education delivery such as language barriers. For example, TDR’s online courses and toolkits for IR were made available in different languages and their partner institutions also provided additional language translations.

Throughout the pandemic, capacity strengthening continued to be a key goal for all three programmes. There were several examples of how they adapted their research focus to aspects of COVID-19 while simultaneously supporting IR capacity strengthening, often by embedded RCS in their COVID-19 research projects. Researchers in AHPSR’s partner institutions worked collaboratively with Ministry of Health staff to undertake data collection for their research alongside COVID-19 community sensitisation. AHPSR issued a call for demand-driven research focused on COVID-19 and facilitated rapid reviews that helped to shape the national response to the pandemic in four countries. HRP included their HRP Alliance partner institutions in >20 LMIC in COVID-19 and sexual and reproductive health and rights research projects, and combined this rapid research response with capacity strengthening.^12^,^14^ Through their partners, the HRP Alliance was also able to provide rapid inputs to the development of clinical management guidelines for COVID-19^15^ and contribute data to the WHO Global Clinical Platform for COVID-19.^16^

## Results and discussion

### Reflections on the process of developing a joint theory of change

The three programmes each have different aims which are closely linked to programme visions (i.e. AHPSR—the use of evidence in decision-making^4^; HRP—sexual and reproductive health and rights for all^5^; TDR—infectious diseases of poverty^6^). Building capacity for IR was not necessarily the main focus for every programme so it was not self-evident that they would be able to find any coherence in their goals and activities. In addition, the programmes often used a ‘learning by doing’ approach which made it difficult to disentangle the activities for ‘doing’ IR from those for ‘building IR capacity’. Some of the participants felt that it may have been beneficial to explore and document these activities in more detail during the discussions. They recognised that the theory of change was one of several tools they used to achieve their own goals. Some activities did not fit within the pathways and, where appropriate, the pathways were adapted to accommodate the activities.

Participants reflected on what had worked well, what could have been done differently, and the benefits of developing a joint theory of change. Despite the complexities of bringing together three programmes with separate funding and mandates to agree on common goals, it became clear that although their programme’s IR capacity strengthening activities were different, they were also complementary. They recognised that ultimately every programme was aiming to reduce inequity and decrease the impact of diseases.

Using a theory of change as a framework helped the entities identify similar activities and develop a joint goal and vision for strengthening capacity for IR. Three common pathways for achieving the programmes’ joint goal of strengthening capacity for IR were identified: skills needed to conduct IR, IR systems and the utilisation of the research outputs. The process of examining programmes’ activities in detail also highlighted gaps that could prevent them from achieving their aims. For example, TDR had traditionally targeted academics for capacity strengthening in IR. They realised they now needed to include national disease control programmes and policy-makers, have greater engagement with institutions conducting IR at the sub-national level, and had adjusted their strategy accordingly.

Participants perceived that external understanding of what goes on within programmes under the WHO ‘umbrella’ was limited. The joint theory of change, with its visual representation of the pathway to achieve impact, was a useful way of summarising and demonstrating coherence in their IR capacity strengthening activities including for the partner institutions that they had in common. Participants felt it was less helpful for identifying areas for future collaboration though these were highlighted during discussions. Although it was developed by three programmes, the joint theory of change has subsequently been used by UNICEF and their partners to increase coherence for their own IR activities.^17^

Our experience of working across several complex global programme to create a theory of change for strengthening capacity in IR has led us to suggest how this process—and the three pathways that emerged—may help other programmes with similar goals (box 1). For example, the three pathways cover (a) the way IR activities are designed, funded and managed (i.e. ‘conduct IR’), (b) strengthening partnerships in IR and individuals’ skills in, for example, research leadership that support IR (‘strengthen IR systems’) and (c) strategies to enhance the knowledge about and uptake of IR research (‘use IR for public health priorities’). Although each theory of change must be designed uniquely for each programme and context, similar pathways may be relevant for consideration by other complex health programmes wishing to develop their own theory of change. We were able to disentangle and understand the themes across all three programme that were relevant for the capacity strengthening goal and it provided a framework for adjusting and tracking activities over time. The practice of using a theory of change is adaptable and transferable and can contribute to improving the effectiveness of other global programmes that aim to strengthen IR capacity, ultimately leading to better decision-making and health improvements.

Box 1 **Suggestions for using a theory of change to guide joint working on research capacity strengthening among complex programmes**

- As a starting point, all the programmes involved should have some commonalities around their vision and goal. The process of developing a theory of change can then help to identify joint activities and coherent pathways to achieve these goals and can catalyse shared learning among the programmes.
- Programmes that wish to come together to strengthen competencies in IR, may benefit from considering our joint theory of change and the pathways that emerged for achieving this goal.
- Use the logic flow provided by a theory of change to help track progress along the pathways from joint activities to outcomes and goal (and ultimately to better health and decision-making) and periodically review and refine the theory of change.
- Administrative support for the theory of change process is helpful, for example, to locate sources of data (e.g., reports, reviews, publications) and to extract progress tracking information. A pragmatic approach is needed for tracking progress so that it does not stall because of a lack of in-depth data or imperfect definitions. The participants representing their various programmes and contributing to the theory of change process need to be selected carefully and, for consistency, the same people need to contribute throughout. They should have in-depth knowledge and an overview of their programmes and adequate time to commit to the process. They also need the active support of their programme leaders since they need to provide information from, and feedback to, other members of their programmes.

## supplementary material

10.1136/bmjph-2023-000029 online supplemental file 1

## Data Availability

Data are available upon reasonable request.
