# Supplementary material for: The process of developing a joint theory of change across three global entities: can this help to make their efforts to strengthen capacity for implementation research more effective?
Source: BMJ Public Health. 2024 Mar 19;2(1):e000029. doi: 10.1136/bmjph-2023-000029 (PMC11816388; doi:10.1136/bmjph-2023-000029)
Supplement: online supplemental file 1 [file bmjph-2-1-s001.pdf]

Supplementary table S1– resources from each programme that were used to complement information provided verbally in the workshop.

TDR

- Implementation Research Training Programmes: A Global Scan Final Report prepared for WHO-TDR
- Annual Report 2019 - Research Capacity Strengthening
- Annual Report 2020 - Research Capacity Strengthening [draft]
- Dako-Gyeke, P., Asampong, E., Afari, E. *et al.* Capacity building for implementation research: a methodology for advancing health research and practice. *Health Res Policy Sys* 18, 53 (2020)
- Go´mez L, Jaramillo A, Halpaap B, Launois P, Cuervo LG, Saravia NG (2019) Building research capacity through "Planning for Success". PLoS Negl Trop Dis 13(8): e0007426.
- Luyckx, V., Reis, A., Maher, D. and Vahedi, M., 2019. Highlighting the ethics of implementation research. *The Lancet Global Health*, 7(9), pp.e1170-e1171.
- IR_Framework 2021-05-28 [draft]
- Allotey, P., Reidpath, D., Certain, E., Vahedi, M., Maher, D., Launois, P. and Ross, B., 2021. Lessons learned developing a massive open online course in implementation research in infectious diseases of poverty in low-and middle-income countries. *Open Praxis*, 13(1), p.127.
- Launois, P., Maher, D., Certain, E. *et al.* Implementation research training for learners in low- and middle-income countries: evaluating behaviour change after participating in a massive open online course. *Health Res Policy Sys* 19, 59 (2021).
- Hooley, C., Baumann, A.A., Mutabazi, V. *et al.* The TDR MOOC training in implementation research: evaluation of feasibility and lessons learned in Rwanda. *Pilot Feasibility Stud* 6, 66 (2020).
- Lessons learnt from a professional development MOOC: engaging culturally and linguistically diverse learners from low- and middle-income countries
- Alonge O, Rao A, Kalbarczyk A, *et al*. Developing a framework of core competencies in implementation research for low/middle-income countries. *BMJ Global Health* 2019;4:e001747. doi:10.1136/bmjgh-2019-001747

HRP

- HRP Annual Report 2019
- HRP Annual Report 2020
- Adanu, R., Bahamondes, L., Brizuela, V. et al. Strengthening research capacity through regional partners: the HRP Alliance at the World Health Organization. Reprod Health 17, 131 (2020). https://doi.org/10.1186/s12978-020-00965-0
- Brizuela, V., Kapilashrami, A., Bonet, M., Khosla, R., Kobeissi, L., Say, L., & Thorson, A. (2021). Sexual and reproductive health and rights of migrants: strengthening regional research capacity. Bulletin of the World Health Organization, 99(5), 402–404. https://doi.org/10.2471/BLT.20.270447

- Compaoré, R., Brizuela, V., Khisa, A.M. et al. 'We always find things to learn from.' Lessons from the implementation of the global maternal sepsis study on research capacity: a qualitative study. BMC Health Serv Res 21, 208 (2021). https://doi.org/10.1186/s12913-021-06195-9
- Thorson A, Aslanyan G, Brizuela V, Perez F, Gómez Ponce de León R, Reeder JC, Serruya SJ, Espinal M, Askew I. Research and research capacity strengthening in the context of an emerging epidemic: Zika virus in Latin America. Int J Gynaecol Obstet. 2020 Jan;148 Suppl 2:1-3. doi: 10.1002/ijgo.13040. PMID: 31975399
- *HRP Alliance for RCS* available at: https://www.who.int/teams/sexual-and-reproductive-health-and-research-(srh)/areas-of-work/human-reproduction-programme-alliance
- *HRP Alliance Special Research Grants* https://www.who.int/reproductivehealth/hrp_alliance/research-grants/en/
- *African Population and Health Research Center* https://aphrc.org/research-capacity-strengthening/
- *Institut de Recherche en Sciences de la Santé* https://hubssr.org/
- *Centro de Pesquisas em Saúde Reprodutiva de Campinas* https://www.cemicamp.org.br/portfolio/hub-oms/
- *Aga Khan University* https://www.aku.edu/mcpk/chs/Pages/Partnerships-and-Collaborations.aspx

AHPSR

- AHPSR 2020 Annual Report
- AHPSR 2019 Annual Report
- Jeanette Vega, Zubin Cyrus Shroff, Kabir Sheikh, Irene Akua Agyepong, Binyam Tilahun, Viroj Tangcharoensathien, Assad Hafeez, Indu Bhushan, Abdul Ghaffar, David Peters, Capacity, committed funding and co-production—institutionalizing implementation research in low- and middle-income countries, *Health Policy and Planning*, Volume 35, Issue Supplement_2, November 2020, Pages ii7–ii8, https://doi.org/10.1093/heapol/czaa120
- Shroff, Z.C., Mancuso, A.B., Sharkey, A. *et al.* Decision-maker led implementation research on immunization: learning from low- and middle-income countries. *Health Res Policy Sys* 19, 68 (2021). https://doi.org/10.1186/s12961-021-00720-2
- Mancuso, A., Ahmed Malm, S., Sharkey, A. *et al.* Cross-cutting lessons from the Decision-Maker Led Implementation Research initiative. *Health Res Policy Sys* 19, 83 (2021). https://doi.org/10.1186/s12961-021-00706-0
- Health Policy and Systems Research in Ethiopia https://ahpsr.who.int/publications/i/item/health-policy-and-systems-research-in-ethiopia
- Health Policy and Systems Research in Mozambique https://ahpsr.who.int/publications/i/item/health-policy-and-systems-research-in-mozambique
- Health Policy and Systems Research in Ghana https://ahpsr.who.int/publications/i/item/health-policy-and-systems-research-in-ghana

- Health Policy and Systems Research in sub-Saharan Africa
- https://ahpsr.who.int/publications/i/item/health-policy-and-systems-research-in-sub-saharan-africa
- Project Brief - Implementation research to strengthen data systems for immunization coverage and equity: Using community health workers to improve data and immunization coverage in Uganda. https://www.ahpsr.org/wp-content/uploads/2021/08/Project-brief-uganda.pdf
- Project Brief - Implementation research to strengthen data systems for immunization coverage and equity: Opportunities to scale-up SIMUNDU: Yogyakarta's innovative electronic immunization registry. https://www.ahpsr.org/wp-content/uploads/2021/08/Project-brief-indonesia.pdf
- Building capacity to take embedded implementation research to scale in Ethiopia https://ahpsr.who.int/newsroom/news/item/04-08-2021-building-capacity-to-take-embedded-implementation-research-to-scale-in-ethiopia
- The bumpy road to better health: how embedded research strengthened health services in Ghana. Geneva: World Health Organization; 2019 (WHO/HIS/HSR/19.2). Licence: CC BY-NC-SA 3.0 IGO. https://apps.who.int/iris/bitstream/handle/10665/331936/WHO-HIS-HSR-19.2-eng.pdf?ua=1
- Ethical considerations for health policy and systems research. Geneva: World Health Organization; 2019. Licence: CC BY-NC-SA 3.0 IGO. https://ahpsr.who.int/publications/i/item/2019-12-02-ethical-considerations-for-health-policy-and-systems-research
- Website: Embedded Research https://ahpsr.who.int/what-we-do/thematic-areas-of-focus/embedded-research
